# Effective Modulation of Ion Mobility through Solid-State Single-Digit Nanopores

**DOI:** 10.3390/nano12223946

**Published:** 2022-11-09

**Authors:** Anping Ji, Bo Wang, Guofeng Xia, Jinjie Luo, Zhenghua Deng

**Affiliations:** 1 School of Mechanical Engineering, Chongqing Three Gorges University, Chongqing 404100, China; 2 Chongqing Engineering Technology Research Center for Light Alloy and Processing, Chongqing 404100, China

**Keywords:** ion mobility, nanopore conductance, surface potential leakage, ion transport

## Abstract

Many experimental studies have proved that ion dynamics in a single-digit nanopore with dimensions comparable to the Debye length deviate from the bulk values, but we still have critical knowledge gaps in our understanding of ion transport in nanoconfinement. For many energy devices and sensor designs of nanoporous materials, ion mobility is a key parameter for the performance of nanofluidic equipment. However, investigating ion mobility remains an experimental challenge. This study experimentally investigated the monovalent ion dynamics of single-digit nanopores from the perspective of ionic conductance. In this article, we present a theory that is sufficient for a basic understanding of ion transport through a single-digit nanopore, and we subdivided and separately analyzed the contribution of each conductance component. These conclusions will be useful not only in understanding the behavior of ion migration but also in the design of high-performance nanofluidic devices.

## 1. Introduction

Modeling and controlling ion transport through nanopores can lead to the manifestation of remarkable physical properties, such as high transport efficiency [1], selectivity [2,3], the ionic-field effect [4], and ionic current rectification [5]. Studies have identified critical gaps in our understanding of nanopores, especially when the pore size transitions from the nanometer to the angstrom scale [6]. In a single-digit nanopore environment with dimensions comparable to the Debye screening length, ion transport typically deviates from the bulk transport mechanism [7,8]. The phenomena of charge overspill [9] and electroneutrality breakdown [10,11] have demonstrated the particularity of the electric double layer in charged nanopores. The electric control of ion transport is of primary importance in the design of novel nanofluidic devices, such as biosensors [12], desalination systems [13], supercapacitors, and energy converters [14,15]. To realize ion transport, nanopores utilize multiple mechanisms, including electrostatic forces, van der Waals forces, and steric interactions [16]. However, such small artificial structures are extremely difficult to manufacture and conduct direct property measurements on because of the lack of high-precision fabrication tools [17]. Moreover, there are many factors affecting ion transport in nanoconfined structures, including the geometry, surface charge, chemical composition, wettability, environmental pH, electrolyte concentration gradient, ion mobility, and electric field strength [18]. Among these factors, the ion mobility is a key parameter determining the performance of nanofluidic equipment, including the gene sequencing resolution [19] and the charging and discharging efficiency of supercapacitors [20]. Accordingly, the effective modulation of ion migration through solid-state single-digit nanopores is key for designing high-performance nanofluidic devices.

Experimentally elucidating the intrinsic mechanisms underlying ion mobility, however, remains a challenge. Ion conductance measurements are regarded as the ideal approach for studying the effects of surface potentials on manipulating the ion mobility of nanoconfined structures. For instance, ion conductance measurements were performed to investigate ion transport in silicon nitride microchannels [21,22]. For a low ratio of the film thickness to pore size, the influence of the access conductance on the entire conductance could not be ignored [23,24,25,26,27]. Accordingly, the breakdown of electroneutrality in nanopores has recently been discovered to significantly affect ion transport [10,11]. In this study, we experimentally investigated the ion mobility of single-digit nanopores from the perspective of ionic conductance. The results showed that the surface potential leakage played a key role in achieving a low ionic concentration, and its contribution to conductance was much higher than that of the surface potential. Furthermore, a model that was developed for estimating the ion mobility performance in the nanopores indicated that the Wien effect significantly enhanced the performance, in comparison to that of the bulk sample.

## 2. Methods

**Figure 1:**
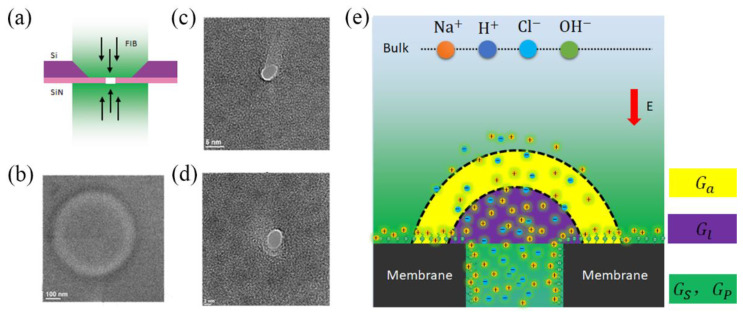
The setup of measuring the ionic currents through nanopores. (**a**) Nanopore fabrication scheme. (**b**) A TEM image of thinned area of silicon nitride film. (**c**) A TEM image of a 3.6 nm nanopore. (**d**) A TEM image of a 2 nm nanopore. (**e**) Schematic illustration of ion migration in a digital nanopore. The salt solution contains four ions, ${\mathrm{OH}}^{-}/{\mathrm{Cl}}^{-}/H^{+}/{\mathrm{Na}}^{+}$. The voltage drives the ions across the nanopore to form a current, the magnitude of which depends on the access, pores, surface charge, electroosmosis, and the potential leakage conductance.

In the present investigation, we experimentally studied the ion dynamics of single-digit nanopores by measuring the concentration dependence of their ionic conductance. Silicon nitride, which is one of the most widely applied semiconductor materials for fabricating nanofluidic devices using mature manufacturing technologies, was selected as the research object. The manufacturing process of single-digit nanopores (D = 2, 3.6, 9, 19 nm) mainly involves chemical deposition, thinning and perforation of the silicon nitride film, as shown in Figure 1. The experimental setup is illustrated in Figure 1e. The chip was assembled into the liquid pool and sealed by means of silicone elastomer gaskets. The transmembrane ionic currents through the nanopore were detected by inserting two electrodes into salt solution with a salt concentration gradient up to seawater salinity. We investigated the coupled effect of the surface potential and the nanoconfined environment in terms of ion mobility by measuring the concentration dependence of the ionic conductance. As the continuum theory could not fully describe the results of many of our experiments, we also conducted a supplementary analysis of the molecular dynamics of the graphene nanopore system (see Supplementary Materials).

## 3. Results & Discussion

### 3.1. Ion Conductance with Solid-State Nanopores

**Figure 2:**
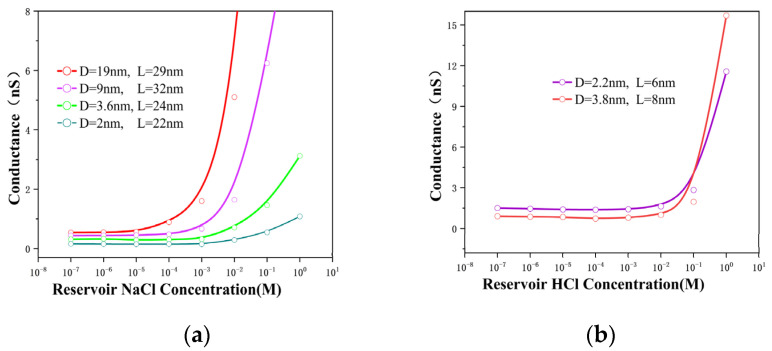
Conductance of HCl and NaCl at concentrations from 10^−7^ M to 1 M. (**a**) The relationship curve between the conductance measured by the nanopore experimental setup with different pore sizes and the concentration of NaCl solution. (**b**) Conductance-concentration curve of HCl.

In this experiment, we measured the conductance of various HCl and NaCl solutions at concentrations ranging from 10^−7^ to 10^0^ M (Figure 2), where diverse power-law relationships were observed between the conductance and ion concentration (G ∝ $n_{0}^{\alpha }$) of these solutions. Our results agreed with those of numerous relevant studies [16,22]. It is well known that ion transport is governed by surface charges at low concentrations, whereas at higher concentrations, its behavior is similar to that in the bulk [28]. As shown in Figure 2a, the conductance of the NaCl solution exhibited a striking saturation at low ionic concentrations. Interestingly, the transition point from the surface-charge-governed to the bulk behavior regimes in the 2 to 3.6 nm nanopores was 1 mM, which was consistent with the results reported for the 2 nm nanochannel [16]. The transition-point value in the 9 nm nanopore decreased to 0.1 mM as the pore size increased. However, the transition point in the 19 nm nanopore was 0.01 mM, which was the same as the conclusion of the microchannel [21]. This low-concentration ion migration behavior in the nanopore was attributed to the enhanced effect of the overlapping electric double layers upon ion diffusion [29]; however, this effect was mitigated with the increase in the pore size. In the bulk behavior regimes, it could be clearly observed that the geometry of the nanopores played a dominant role in ion transport. In the comparative experiment with the HCl solution, the conductance of the 2.2 nm nanopore was greater than that of the 3.8 nm nanopore for ion concentrations of up to 100 mM and a relatively similar film thickness (Figure 2b). As there was no surface charge below pH = 2 due to the full protonation of the channel surface, the conductance of the 3.8 nm nanopore exceeded that of the 2 nm nanopore. To further understand the causes of this phenomenon, it is necessary to study the role of each ion conductance component separately.

### 3.2. Theoretical Conductance Model with Electric Potential Leakage

It has been demonstrated in both experiments and simulations [28] that the net charge of the solution in single-digit nanopores is not sufficient to maintain the electroneutral condition, which exhibits phenomena such as charge overspill [30], end effects [9], and electroneutrality breakdown [10,11]. It is worth noting that the lack of electroneutrality in the pore region does not indicate a failure of electroneutrality of the entire system. The electrical neutrality of the entire system is maintained by a combination of the net charge of the solution in the pore region and the net charge in the reservoir. The surface charge potential leaks into the reservoir, resulting in an inadequate net charge inside the pores, which is the physical source of the lack of a net charge. However, earlier studies did not take this factor into account, and this leakage cannot be ignored in single-digit nanopores, due to the strengthening of counterion transport and the weakening of co-ion transport.

The total conductance of the nanopore comprises two main components: electrophoresis conductance (*G_ph_*) and electroosmotic conductance (*G_eo_*), which is affected by the ionic migration-driven water transport. Moreover, as shown in Figure 1b, the electrophoresis conductance consists of four parts: access conductance (*G_a_*), nanopore conductance (*G_p_*), surface conductance (*G_s_*), and electric-potential leakage conductance (*G_l_*). Thus, the entire conductance (*G_0_*) of a charged nanopore can be theoretically predicted by combining these parts into the following equation, suggested by earlier works [29,31,32,33]:

(1) $$G_{0}=\underset{i}{\sum }F\left\{n_{i}^{+}(\mu_{i}^{+}+\mu_{eo}){\left(\sqrt{1+{\tilde{\sigma }}^{2}}+\tilde{\sigma }\right)}^{1-\alpha }+n_{i}^{-}(\mu_{i}^{-}-\mu_{eo}){\left(\sqrt{1+{\tilde{\sigma }}^{2}}-\tilde{\sigma }\right)}^{1-\alpha }\right\}{\left(\frac{L}{\pi R^{2}}+\frac{1}{2R}\right)}^{-1}$$

where $n_{i}$ and $u_{i}$ are the concentration and electrophoretic mobility of an ion of the ith species, respectively; *R* and *L* are the radius and the length of a nanopore, respectively; *F* is the Faraday constant; $\;\mu_{eo}$ is the electroosmotic mobility; α is the fraction of the surface electric potential that leaks out of the pore; and $\tilde{\sigma }$ is a dimensionless coefficient. Here, $\tilde{\sigma \;}$ is the ratio between the net charge concentration required in the nanopore for electroneutrality and the charge concentration of the bulk solution. For a 1:1 electrolyte and homogeneous charge distribution in a cylindrical nanopore, $\tilde{\sigma }\;$ can be written as $\;\tilde{\sigma }=-\frac{\sigma }{FRn_{0}}$, where $n_{0}$ and *σ* are the nanopore concentration and surface charge density, respectively.

**Figure 3:**
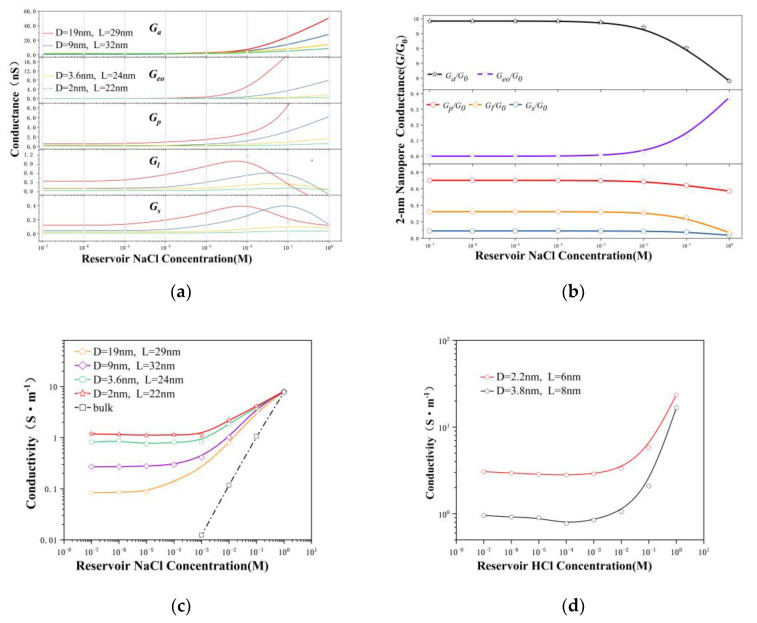
Ionic conductance and conductivity. (**a**) Access conductance (*Ga*), nanopore conductance (*Gp*), surface conductance (*Gs*), electric potential leakage conductance (*G_l_*), and electroosmotic conductance (*Geo*) of NaCl solution. (**b**) In 2 nm nanopore, ratio of $\;G_{a},G_{p},G_{l},G_{s},G_{eo}$ to the total ionic conductivity (*G*_0_) as a function of reservoir concentration. (**c**) Ionic conductivity of Nacl solution. (**d**) Ionic conductivity of Nacl solution.

By combining Equation (1) and the experimental measurement data, the relationship between different types of conductance and concentrations can be obtained (Figure 3). For the electrolyte with an ionic concentration lower than 10 mM, the conductance values followed this sequence: $G_{a}>G_{p}>G_{l}>G_{s}>G_{eo},$ and this order of the conductance depends on the aspect ratio geometries of the nanopores as well. The increase in the bulk concentration ($n_{0}$) also leads to a power-law increase in the conductance values ($G_{a},G_{p},G_{l},G_{eo},G_{s,}\propto n_{0}^{\alpha }$). At the same time, we observed that the conductance of each part increased as the aperture increased. The electrophoretic conductance (*G_ph_*) can be written as:

(2) $$G_{ph}^{-1}=G_{a}^{-1}+{\left(G_{S}+G_{P}+G_{l}\right)}^{-1}$$

The access conductance was revealed to be at least one order of magnitude higher than that of the other parts; consequently, the access part presented the least hindrance to ion transport (Figure 3a). The access conductance of the 2 nm nanopore was 16–20 times the total conductance ($G_{0}$), as shown in Figure 3b. The entrance part with a good conductivity was connected in series with the nanopore part with a low conductivity, causing the ions in the high-concentration queue to wait until they could pass through the nanopore. This behavior was exactly the cause of the concentration polarization, which was consistent with the MD conclusions (see Figure S1). Ion transport behavior through the nanopores (L~D) on the film differs from that in the nanopores (L >> D) and is even more different from bulk activity. In Figure 3a, we also observe that the conductance variation is more prominent when the D/L ratio is larger.

Figure 3b shows that the overall conductance of the 2 nm nanopore ($G_{nc}=G_{S}+G_{P}+G_{l}$) at a concentration below 10 mM was approximately equal to the total conductance (*G_0_*). According to Equation (2), *G_nc_* is the decisive component of the ion transport behavior. Half of the nanopore conductance is determined by the surface conductance and potential leakage conductance, suggesting that ion dynamics within charged nanopores are still governed by the surface charge in low-concentration electrolytes. This phenomenon is consistent with earlier reports [4,21,34,35]. At the same time, we also observed that $G_{l}$ was approximately six times $G_{s}$ at concentrations below 100 mM, implying that $G_{l}$ played a key role in the surface-charge-governed regime. Accordingly, Figure 3a shows that the concentrations corresponding to the peaks of the surface conductance and potential leakage conductance decreased along with the increasing pore size (10 mM in 19 nm pores and 100 mM in 2 nm pores). In contrast, the concentration corresponding to the peak of the electroosmotic conductance started to increase rapidly, leading to inconsistent transition points of the total conductance (*G_0_*) as the aperture changed.

To further understand the ion transport behavior, the conductivity obtained after removing the geometric factors was investigated. We can simply express the conductance as $G=\kappa {\left(\frac{L}{\pi R^{2}}+\frac{1}{2R}\right)}^{-1}$, where κ is the conductivity of the electrolyte inside the pore. The conductivity can be written as:

(3) $$\kappa =\underset{i}{\sum }F\left\{n_{i}^{+}(\mu_{i}^{+}+\mu_{eo}){\left(\sqrt{1+{\tilde{\sigma }}^{2}}+\tilde{\sigma }\right)}^{1-\alpha }+n_{i}^{-}(\mu_{i}^{-}-\mu_{eo}){\left(\sqrt{1+{\tilde{\sigma }}^{2}}-\tilde{\sigma }\right)}^{1-\alpha }\right\}$$

Figure 3c,d depict the power-law relationship between the conductivity and concentration (κ ∝ $\;n_{0}^{\alpha }$), revealing that the conductivity approached the bulk behavior as the pore size of the nanopore increased to 19 nm. It is worth noting that a smaller nanopore size leads to a higher conductivity, which is determined by the carrier concentration and mobility.

### 3.3. The Surface Charge and Ensemble-Averaged Concentration inside the Pore

**Figure 4:**
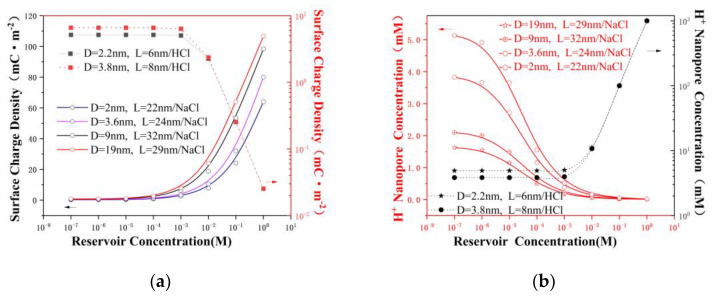

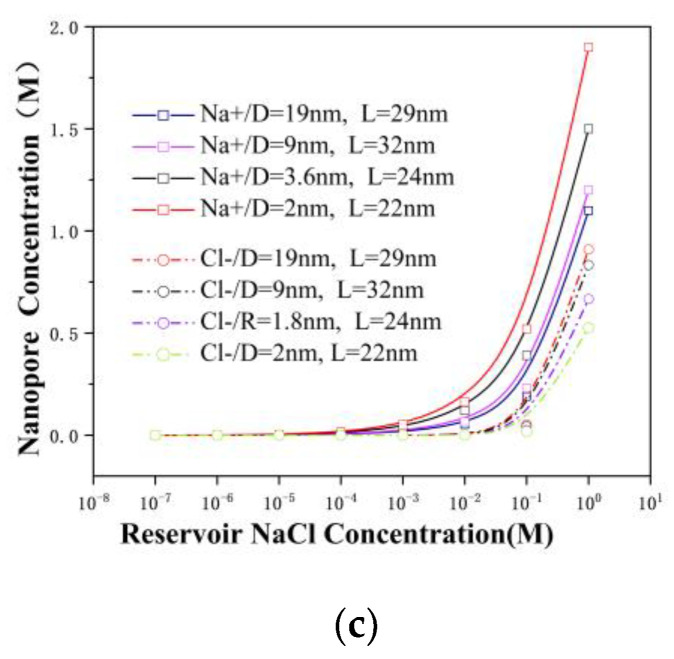
Ionic concentration inside the nanopore. (**a**) Surface charge density inside the nanopore for NaCl/HCl solutions. (**b**) H^+^ concentration inside the nanopore. (**c**) Na^+^/Cl^-^ concentration inside the nanopore.

The ion concentration and surface charge density were estimated using the model from reference [16], as shown in Figure 4. The four main types of ions that are responsible for the transport and maintenance of neutrality in the nanopore were ${\;\mathrm{OH}}^{-}/{\mathrm{Cl}}^{-}/H^{+}/{\mathrm{Na}}^{+}$, and the corresponding concentrations were *n*_OH^−^_/*n*_Cl^−^_/*n*_H^+^_/*n*_Na^+^_, where $n_{ion\;}$ is the ensemble-averaged concentration inside the pore (access [36], surface charge potential leakage [29], and nanopore, as shown in Figure 1b). Because the experiment was completed under ambient conditions, the formation of $H^{+}$/${\mathrm{OH}}^{-}$ was caused by CO_2_ absorption, which caused the pH of the electrolyte solution to reach approximately 5 [16,22]. For the NaCl solution, the increased ${\mathrm{Na}}^{+}$ concentration inside the nanopore led to a decrease in the $H^{+}$ concentration, which reduced the protonation of the surface and increased the surface charge density. In the low-concentration area of the NaCl solution, $H^{+}$ acted as the main carrier; at the same time, the concentration of smaller nanopores was higher, which led to a similar situation in terms of the conductivity. At ~10^−3^ M, although the concentration in the nanopore and the surface charge also increased quickly, the ions were always in a state of depletion to maintain electrical neutrality, and the conductance of the NaCl solution exhibited a striking saturation. Furthermore, the $n_{Na^{+}}$ value increased along with the increasing pore size, whereas the opposite was exhibited for $n_{Cl^{-}}$ (Figure 4c and Figure S2). Considering that the surface charge enhanced the counterion transport while also reducing the co-ion transport, such a result was expected and consistent with the results in Figure 3a. However, in the HCl solution, because the surface was almost completely protonated when the pH was lower than 2, the $n_{H^{+}}$ value was completely determined by the bulk concentration (Figure 4a,b).

### 3.4. Ion Mobility

**Figure 5:**
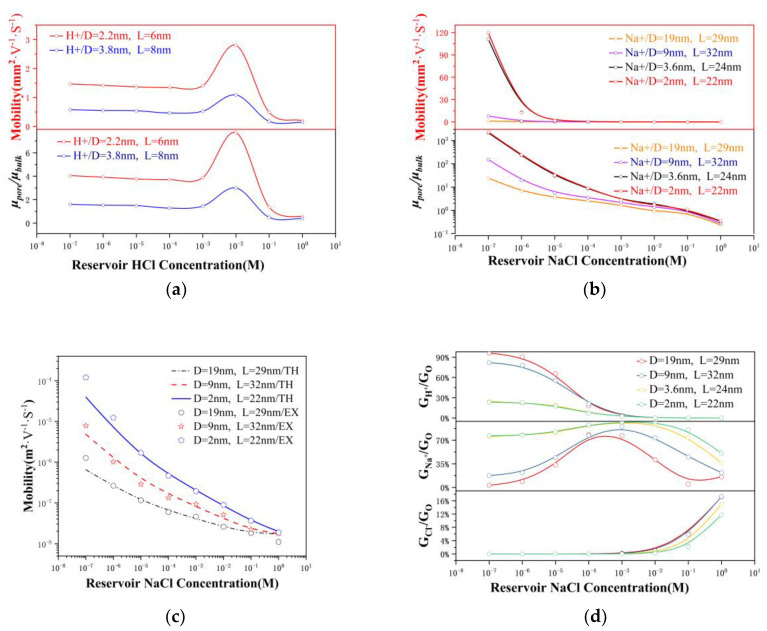
Ion mobility and selectivity. In (**a**,**b**), ion mobility and ratio of ionic mobility inside the nanopore ($\mu_{pore}$) to the corresponding bulk mobility ($\mu_{bulk}$ ) as a function of reservoir concentration. (**c**) Mobility-concentration curves from various experiments and the mobility theory given in Equation (4). (**d**) Ion selectivity for the nanopore. Comparisons between the contributions of different ions to conductance.$\;G_{H^{+}},\;G_{Cl^{-}},\;\mathrm{and}\;G_{Na^{+}}$ are the H^+^, Cl^−^, and Na^+^ conductances, respectively.

Figure 5a illustrates that up to a bulk concentration of 10^−3^ M, the proton mobility inside the 2.2 nm nanopore was discovered to be four times the bulk proton mobility $(\mu_{H^{+}}^{bulk}=\mu_{H^{+}}^{\infty }=3.627\times 10^{-7}\;m^{2}\cdot V^{-1}\cdot s^{-1},H^{+}$ in infinite-diluted solutions), which was consistent with the conclusion of the 2 nm nanochannel [16]. Following the formation of hydrated ions, proton transport was then realized via tunneling. Therefore, ion mobility was determined by the rearrangement speed of the water molecules [17,37]. Using molecular dynamics to calculate the potential of water molecules in a 2 nm nanopore system, it was revealed that the higher the voltage, the stronger the rearrangement ability of the water molecules (see Figure S1). This caused the proton mobility in the nanopore to surpass that of the bulk material. Since the surface charge was almost negligible when the pH value was 2, the overlapping electric double layer had no electrostatic effect on the counterions, enabling the ion mobility to peak. After the ion concentration exceeded 10^−2^ M, there was no repulsive effect from the surface charges on the ${\mathrm{Cl}}^{-}$ species, allowing them to participate in the current transport mechanism. As a result, the rearrangement of water molecules was weakened and the ionic mobility was compromised. Since larger nanopores have a greater surface charge density and a strengthened overlapping electric double layer has a stronger effect on ion transport, a decrease in mobility was encountered as the pore size increased $(\mu_{3.8nm}<$ $\mu_{2.2nm}$). Figure 5b illustrates that the Na^+^ mobility in the NaCl solution was higher than the maximum bulk mobility ($\mu_{{\mathrm{Na}}^{+}}$ in infinite-dilution is $5.19\times 10^{-8}\;m^{2}\cdot V^{-1}\cdot s^{-1}$) at ~10^−2^ M. At 1 M, the mobility was reduced by approximately 65% from that of the bulk value, a similar result as those reported for graphene nanopores [38] and carbon nanotubes [39]. Similar to the HCl solution, the ion mobility decreased as the pore size increased. As the electric field strength inside the nanopore (>2.2 × 10^7^ V m^−1^ in this experiment) was higher than the critical electric field of the Wien effect (10^7^ V m^−1^), the acceleration of the ion mobility under the Wien effect was predictable [39]. This phenomenon provides new insights for the design of supercapacitor nanoporous electrodes and nanopores for biosensor detection applications with time resolution modulation features.

In a nanopore system containing a 1:1 electrolyte under environmental conditions, the variation in the ion mobility inside the pore can be expressed as:

(4) $$\mu_{ion}^{nanopore}=\frac{1}{3}\mu_{ion}^{bulk}{\left(1+\frac{\sigma }{FRn_{bulk}}\right)}^{0.5-\alpha }$$

where $\mu_{ion}^{nanopore}$ and $\mu_{ion}^{bulk}$ are the ion mobilities of ions within the nanopore region and in bulk liquid, respectively, and α is the fraction of the surface electric potential that leaks out of the pore. This equation demonstrates that the ion mobility inside the nanopore is determined by the ionic bulk mobility, bulk concentration, nanopore geometry, and surface charge. Figure 5c illustrates the mobility-concentration curves resulting from our experiments. Most of the experimental data points can be described well using the mobility theory in Equation (4).

### 3.5. Ion Selectivity

There is a significant difference in the ion exchange ability of the pore to pass/reject the two ion types, and this characteristic can be defined as the ion selectivity of the pore [40,41]. According to Equation (3), we can obtain the relationship between the conductance contributed by different ions and the total conductance, as shown in Figure 5d. Ion selectivity is defined as [28]:

(5) $$S_{ion}=\frac{G_{ion}}{G_{total}}$$

where $G_{ion}$ and $G_{total}$ are the conductance of a particular ion and the total system conductance, respectively. At concentrations below 10^−5^ M, the contribution of H+ species to conductance is dominant. From 10^−5^ to 10^−3^ M, the conductance was mainly driven by sodium ions and $H^{+}$. At concentrations greater than 10^−3^ M, the conductance resulted from the combined action of ${\mathrm{Na}}^{+}$ and ${\mathrm{Cl}}^{-}$. Notably, the Na+ selectivity in the 2 nm nanopore reached a peak of 97% at 10^−2^ M as the bulk concentration increased; however, in the 19 nm nanopore, it only reached 77%. Interestingly, the ${\mathrm{Cl}}^{-}$ selectivity was always less than 20% and increased as the pore size increased, contrary to the case of chloride ions. This phenomenon was assigned to the surface charges, which repelled the ions of the co-ion (${\mathrm{Cl}}^{-}$) and simultaneously attracted the counterions (Na+), thereby enhancing the Na+ selectivity. At concentrations above 10^−2^ M, the Na+ selectivity decayed, while the phenomenon of electroosmotic flow started to manifest and was rapidly enhanced.

## 4. Conclusions

In this study, the monovalent ion dynamics of single-digit nanopores were experimentally investigated from the perspective of ionic conductance. The conductance transition point was discovered to change with the pore size of the nanopore. In the single-digit nanopores, the overlapping electric double layers strengthened the ionic transport behavior and increased the conductivity of the nanopore. To evaluate the specific role of the potential leakage conductance, we subdivided and separately analyzed the contribution of each conductance component. After establishing a reconstructed theoretical model, the access conductance, nanopore conductance, surface conductance, and potential leakage conductance were investigated in detail. The results showed that the factor of surface potential leakage played a key role in achieving a low ion concentration, and its overall contribution to conductance was much higher than that of the surface potential. At the same time, the ion concentration in the pores was significantly higher than that in the bulk, which is an important indicator of enhanced conductivity in a nanoconfined environment. A model was also established to investigate the ionic mobility in the nanopores, revealing that the Wien effect manifested under a high electric field strength. This discovery notably contributes to the fundamental understanding of the intrinsic mechanisms underlying ion migration, and it is prospected to inspire many new technologies in the field of high-performance nanofluidic devices.

## Data Availability

Not applicable.

## Supplementary Materials

The following supporting information can be downloaded at: https://www.mdpi.com/article/10.3390/nano12223946/s1. Figure S1: Water and ions passing through graphene nanopore. Figure S2: Ionic concentration inside the 2-nm nanopore. References [4,11,16,22,25,27,28,31,32,33,36,38,42,43,44,45,46,47,48,49] are cited in the supplementary materials.
