# Supplementary material for: Effective Modulation of Ion Mobility through Solid-State Single-Digit Nanopores
Source: Nanomaterials (Basel). 2022 Nov 9;12(22):3946. doi: 10.3390/nano12223946 (PMC9695415; doi:10.3390/nano12223946)
Supplement: Supplementary file 1 [file nanomaterials-12-03946-s001.zip › nanomaterials-1989129-supplementary.pdf]

Supporting Information

Effective Modulation of Ion Mobility through Solid-State Single-Digit Nanopores

Anping Ji, [a, b] Bo Wang [a] Guofeng Xia [a] Jinjie Luo [a, b] Zhenghua Deng [a, b]

a. School of Mechanical Engineering, Chongqing three gorges university, Chongqing, 404100. E-mail: ji-anping@sanxiau.edu.cn

b. Chongqing Engineering Technology Research Center for Light Alloy and Processing*, Chongqing, 404100*

1. Experiment detail

First, a 100 nm thick Si_3N_4 membrane was grown on the silicon wafer by low pressure chemical vapor deposition. Then, a window on the other side of the wafer was opened using wet etching process to expose the silicon nitride thin film. After the etching process, the silicon nitride film was exposed to a focused ion beam with high energy to reduce the film thickness to 20 nm. At the last step, a nanopore can could be drilled by the transmission electron microscope (TEM) beam operated at 300kV. The fabricated nanopore size could be tuned using the electron beam with low intensity. First, it is necessary to clean the wafer on which the nanopore is fabricated with piranha solution to remove any contaminants. The silicon nitride film with 2-nm nanopores divides the liquid pool into aqueous cis. and trans. chambers. Ag/AgCl electrodes immersed on both sides of the pore are connected to a patch clamp amplifier (HEKA EPC 10 USB, HEKA Instruments) to measure the ionic current with picoampere sensitivity. The device was placed in a double faraday cage to reduce electrical noise. We add the degassed and filtered salt solution to the liquid pool, and then wait until it reaches a stable state before starting the experiment. By sweeping the voltage from -500mV to 500mV at a scan rate of 100 mV per 2 seconds, we can measure many sets of current data. During the measurement, we increase the bulk concentration from low to high (10^{-7} M ~ 10^{0} M).

2. Molecular dynamics

In this study, ion concentration polarization layer is investigated with molecular dynamic (MD) simulation. A highly efficient MD package GROMACS was performed to study the influence of electrical field, ion concentration and diameter of nanopore.

The system selects a cube box as the research unit, the box size is: $L_x=5.28$ *nm,* $L_y=5.39$ *nm,* $L_z=10.60$ *nm* .The conditions is electric field strength E=0V nm^{-1}, 0.3V nm^{-1}, 0.5V nm^{-1}, 1V nm^{-1}, the NaCl concentration 0.1M,0.5M,1M, and nanopore diameter D=2nm, where $z=5nm$ corresponds to the graphene sheet. The bond length between carbon atoms is 0.142 nm, and the box is filled with TIP3P model water molecules. The system model is completed by the cooperation of the software GROMACS 5.02 and VMD(Figure S1).

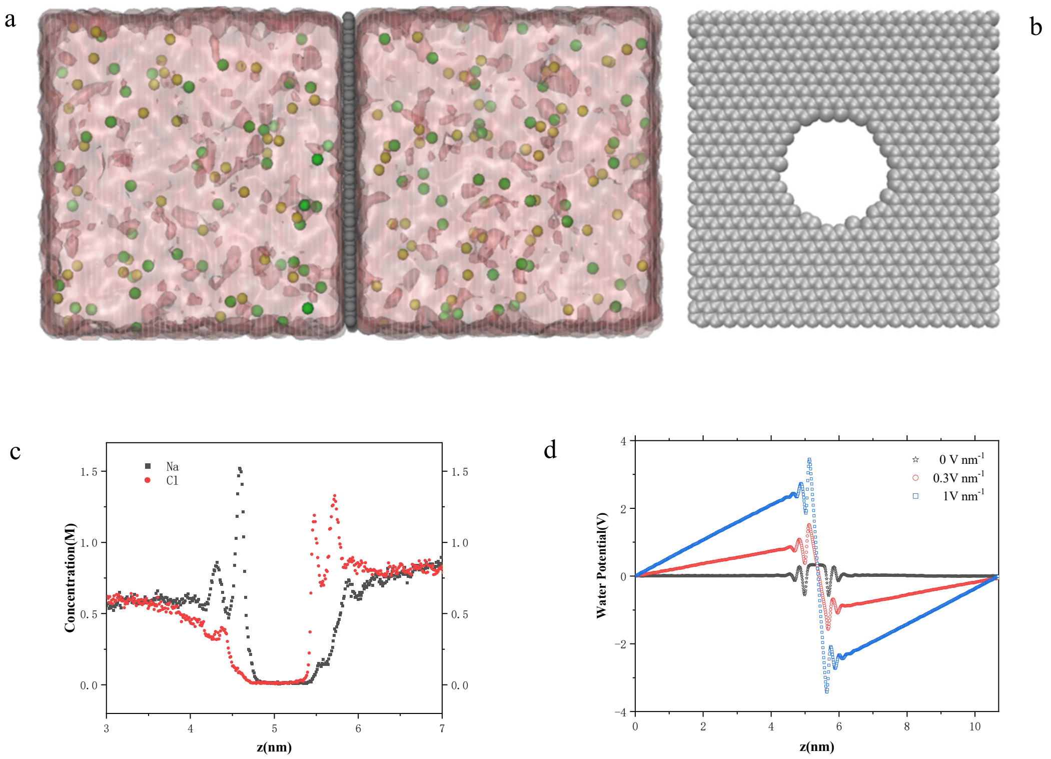

Figure S1. Water and ions passing through graphene nanopore. (a). Schematic diagram of water and ions passing through graphene nanopore model.(b).graphene nanopore； (c). the axial distribution of ion concentration from molecular dynamics results. (d). Average axial electrostatic potential for H_2O in a MD model with 2-nm nanopore. The stronger the electric field, the stronger the ability to rearrange water molecules.

All parameter settings refer to earlier reports[38,31]. After the experiment is over, a comparison with some of the reported results verifies the feasibility of the experiment. The system temperature is stable at 300 K. The pressure is at 1 atmosphere. The temperature coupling method is the V-rescale method, and the pressure coupling method is the Parrinello-Rahma method. The simulation uses the OPLS all-atom force field, and the van der Waals interaction between ions is calculated by the Lennard-Jones (LJ) model. The cutoff radius is 1.0nm, while the Coulomb electrostatic interaction is calculated by the PME (particle-mesh Ewald) method. The cutoff distance is 1.0 nm. In order to drive the ions to move directionally in the system, a uniform electric field is applied in the Z direction. The atoms along the four edges of graphene are fixed. The time interval of each step of the system simulation is 2 fs, and the total number of steps is 5 million steps. The data storage frequency is 0.2 ps, and the total running time of the system is 10 ns.

In Figure S1, the molecular dynamics model and partial results are shown. Figure S1C shows the polarization of the ion concentration at both ends of the nanopore, which is consistent with our conductance calculation. When the concentration is increased to 1M, the higher entrance conductance causes ions to queue up into the nanopore. Figure S1d shows that the ability of water molecules to rearrange increases with increasing electric field strength.

3. Theoretical model for conductance

When many experiments study ion dynamics in micron-sized channel, and conductance

measurement (for neutral pores) is estimated as[42,43]

$$G_P = \sum_i Fn_i\,\mu_i \left(\frac{L}{\pi R^2}\right)^{-1}. \quad (1)$$

However, the conductance (G_p) only is a reasonably accuracy for channels (L ≫ R) with a very large aspect ratio L/R, where the access resistance is negligible. When the ratio of film thickness to pore size is small (L ~R), the influence of entrance resistance on system resistance cannot be ignored [25–27,32,33]. Theoretically, the access conductance(G_a) was derived by Hall[44] and can be expressed as $G_a = \sum_i Fn_i\,u_i 4\mathrm{R}$. Ionic conductance of ions can be theoretically predicted through a combination of access resistance and pore resistance as suggested by earlier works:[26,27,36,38]

$$G_0 = \sum_i Fn_i\,\mu_i \left(\frac{L}{\pi R^2} + \frac{1}{2R}\right)^{-1}. \quad (2)$$

For the charged nanopore, the surface conductance make an additional contribution to the entire conductance, as nanopores requires counterions to screen the surface charge and maintain electrical neutrality[4,45]. The surface conductance(G_s) can be expressed as $G_a = \mu_s \frac{2\pi\sigma\mathrm{R}}{L}$, where μ_s is the mobility of counterions of a charged pore surface[11]. The entire conductance(G_0) can be written as $G_0^{-1} = G_a^{-1} + (G_S + G_P)^{-1}$. By combining equations 1 and 2, the total conductance can be expressed as[28]

$$G_0 = \sum_i Fn_i\,\mu_i \sqrt{1+\tilde{\sigma}^2} \left(\frac{L}{\pi R^2} + \frac{1}{2R}\right)^{-1}. \quad (3)$$

Here, the value of $\tilde{\sigma}$ is the ratio of the net charge concentrations required in the pore and the charge concentration of the bulk solution, which is a dimensionless coefficient. For the experimental system where a 1:1 solution is placed in a cylindrical pore with homogeneous charge distribution, $\tilde{\sigma}$ can be written as $\tilde{\sigma} = -\frac{\sigma}{FRn_0}$.

A large number of experiments have shown the failure of the principle of electrical neutrality inside the nanopore region, such as charge overspill, end effects, the surface-electric-potential Leakage and electroneutrality breakdown, but the entire nanopore system remains electroneutrality [11,28,46–48]. The reason for these phenomena is that the surface charge potential leaks into the reservoir and the net charge inside the pores is insufficient. This potential leakage is different from the effect of surface charges, which enhances the counterion transport and weakens the co-ion transport. Taking the electric potential leakage conductance (G_l) into consideration, the electrophoretic conductance (G_{ph}) can be expressed as

$$G_{ph} = \sum_i F\left\{n_i^+\mu_i^+\left(\sqrt{1+\tilde{\sigma}^2}+\tilde{\sigma}\right)^{1-\alpha} + n_i^-\mu_i^-\left(\sqrt{1+\tilde{\sigma}^2}-\tilde{\sigma}\right)^{1-\alpha}\right\}\left(\frac{L}{\pi R^2} + \frac{1}{2R}\right)^{-1} \quad (4)$$

where α is the fraction of the surface-electric potential that leaks out of the pore [28]. The total conductance of the nanopore should be composed of two parts: electrophoresis conductance (G_{ph}) and electroosmotic conductance (G_{eo}) affected water transport driven by the ionic migration. Then, the entire conductance (G_0) can be written as [4,28]

$$G_0 = \sum_i F\left\{n_i^+(\mu_i^+ + \mu_{eo})\left(\sqrt{1+\tilde{\sigma}^2}+\tilde{\sigma}\right)^{1-\alpha} + n_i^-(\mu_i^- - \mu_{eo})\left(\sqrt{1+\tilde{\sigma}^2}-\tilde{\sigma}\right)^{1-\alpha}\right\}\left(\frac{L}{\pi R^2} + \frac{1}{2R}\right)^{-1}. \quad (5)$$

4. Theoretical model for the ensemble averaged concentration inside the nanopore

For a given surface charge density σ, the ion concentration inside the nanopore must satisfy a certain relationship due to the quasi-electroneutrality condition, as suggested by earlier works [16]:

$$\frac{\sigma}{eR} = n_{OH^-} + n_{Cl^-} - n_{H^+} - n_{Na^+}. \quad (6)$$

$n_{OH^-}/n_{Cl^-}/n_{H^+}/n_{Na^+}$ are used to represent the concentration of $OH^-/Cl^-/H^+/Na^+$, which are different from bulk concentration n_0. By using the Donnan equilibrium condition to relate electric potentials and Poisson-Boltzmann theory, we can get such a relationship:

$$\frac{n_{ion}}{n_{bulk}} = \exp\langle\frac{-e}{kT}(\phi_{ion} - \phi_{bulk})\rangle. \tag{7}$$

For silicon nitride film, the surface charge is related to the PH value, and its value can be expressed as

$$\sigma = \frac{\sigma_0 K_d}{K_d + n_{H^+}} \tag{8}$$

σ_0 is the maximum possible charge density(-1.28C · m^{-2} in this experiment), and Kd is the equilibrium dissociation constant(equal to 10^{-6} M^{10}) [16,22].

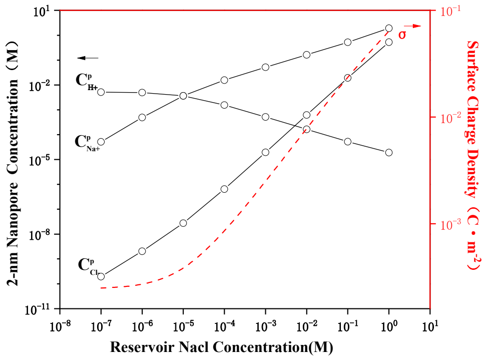

Figure S2. Ionic concentration inside the 2-nm nanopore. Ionic concentration inside the nanopore and surface charge density for NaCl solutions. Nanopore ionic concentration and surface charge density is increases with increasing reservoir ion concentration.

5 Electroosmotic and ion mobility

Electroosmotic mobility. The electroosmotic mobility derived by Biesheuvel and Bazantusing SC theory and the Donnan equation can be expressed as [28,49]

$$\mu_{eo} = \frac{-\sigma R}{4\eta} \tag{9}$$

where η is the viscosity of the solution. η can be obtained from the Stokes equation.

Ion mobility. In this paper, we assume that the transport of different ions in the nanopore has a similar relationship with the transport of bulk behavior. For fully dissolved sodium chloride solution, ionic mobility in a bulk solution can be obtained, as suggested by earlier works:

$$\mu_{bulk} = \frac{\mu_{bulk}}{1 + \frac{0.508 * \sqrt{I_Z}}{3.29 * \alpha\sqrt{I_Z}}} \tag{10}$$

The above equation applies to ambient temperature at 25°C, $\mathbf{I_z} = \frac{1}{2}\sum_j n_j^2$ is the ionic strength, and α is an adjustable parameter related to the ion size (Cl-, a ≈ 0.3 nm; Na+, a ≈ 0.4 nm) [16].
